# Do Double-Expressor High-Grade B-Cell Lymphomas Really Need Intensified Treatment? A Report from the Real-Life Series of High-Grade B-Cell Lymphomas Treated with Different Therapeutic Protocols at the Institute of Oncology Ljubljana

**DOI:** 10.3390/biomedicines12020275

**Published:** 2024-01-25

**Authors:** Lučka Boltežar, Samo Rožman, Gorana Gašljević, Biljana Grčar Kuzmanov, Barbara Jezeršek Novaković

**Affiliations:** 1 Division of Medical Oncology, Institute of Oncology, Zaloška Cesta 2, 1000 Ljubljana, Slovenia; lboltezar@onko-i.si; 2 Medical Faculty, University of Ljubljana, Vrazov Trg 2, 1000 Ljubljana, Slovenia; 3 Pharmacy, Institute of Oncology, Zaloška Cesta 2, 1000 Ljubljana, Slovenia; samorozman@gmail.com; 4 Department of Pathology, Institute of Oncology, Zaloška Cesta 2, 1000 Ljubljana, Slovenia; ggasljevic@onko-i.si (G.G.); bkuzmanov@onko-i.si (B.G.K.)

**Keywords:** double-expressor lymphoma, double-hit lymphoma, R-CHOP, R-DA-EPOCH, IPI score

## Abstract

High-grade B-cell lymphomas with *MYC* and *BCL2* and/or *BCL6* rearrangements are known for their aggressive clinical course and so are the ones with MYC and BCL2 protein overexpression. The optimal therapy for these lymphomas remains to be elucidated. A retrospective analysis of all diffuse large B-cell lymphomas and high-grade B-cell lymphomas with *MYC* and *BCL2* and/or *BCL6* rearrangements diagnosed between 2017 and 2021 at the Institute of Oncology Ljubljana, Slovenia, has been performed. Only patients with double-expressor lymphoma (DEL), double-hit lymphoma (DHL), or triple-hit lymphoma (THL) were included. Demographic and clinical parameters were assessed, as well as progression-free survival (PFS) and overall survival (OS). In total, 161 cases out of 309 (161/309; 52,1%) were classified as DEL. Sixteen patients had DHL, *MYC*/*BCL2* rearrangement was observed in eleven patients, and *MYC*/*BCL6* rearrangement was observed in five patients. Five patients were diagnosed with THL. Out of 154 patients (according to inclusion/exclusion criteria) included in further evaluation, one-hundred and thirty-five patients had double-expressor lymphoma (DEL), sixteen patients had DHL, and three patients had THL. In total, 169 patients were treated with R-CHOP, 10 with R-CHOP and intermediate-dose methotrexate, 19 with R-DA-EPOCH, and 16 with other regimens. The median follow-up was 22 months. The 5-year OS for the whole DEL group was 57.1% (95% CI 45.9–68.3%) and the 5-year PFS was 76.5% (95% CI 72.6–80.4%). The log-rank test disclosed no differences in survival between treatment groups (*p* = 0.712) while the high-risk international prognostic index (IPI) carried a significantly higher risk of death (HR 7.68, 95% CI 2.32–25.49, *p* = 0.001). The 5-year OS for DHL patients was 32.4% (95% CI 16.6–48.2%) while all three TH patients were deceased or lost to follow-up. Our analyses of real-life data disclose that the R-CHOP protocol with CNS prophylaxis is a successful and curative treatment for a substantial proportion of DEL patients.

## 1. Introduction

The 2016 revised WHO classification of lymphoid neoplasms brought mandatory molecular testing of diffuse large B-cell lymphomas (DLBCLs) into routine clinical practice [1]. Large B-cell lymphomas with a *MYC* rearrangement, along with B-cell lymphoma 2 (*BCL2*) and/or B-cell lymphoma 6 (*BCL6*) rearrangements, are being classified as high-grade B-cell lymphomas (HGBLs) and are frequently referred to as double-hit (DHL) and triple-hit lymphomas (THL), respectively. However, if rearrangements are not found and only immunohistochemical (IHC) overexpression of MYC and BCL2 proteins is present, then the lymphoma is classified as double-expressor DLBCL (DEL) [1]. The IHC thresholds of ≥40% for MYC and >50% for BCL2 are currently used in clinical practice to define DEL [1]. According to some studies, MYC and BCL2 overexpression in the absence of chromosomal translocations are attributable to gene amplifications and post-translational processes in malignant cells [1,2]. Moreover, up to 20% of patients with DHL do not demonstrate an overexpression of *MYC* and *BCL2* at a protein level; it seems that this population of patients may have improved outcomes compared to those with DHL with concurrent dual protein expression [2]. Based on retrospective studies, *MYC* rearrangement is usually associated with inferior overall survival (OS); however, some authors reported similar outcomes between patients with *MYC* amplification, or other rearrangements, and those without them [2]. It has also been shown that patients with *MYC* and *BCL2* and/or *BCL6* gene extra copies could have poorer prognosis [3]. Some authors describe the diffuse strong staining of BCL2, termed the superexpression of BCL2, that carries a worse event-free survival (EFS) and OS [4]. Recently, the renewed WHO classification removed *BCL-6* from the classification, leaving only rearrangements of *MYC* and *BCL2* to define double-hit lymphomas [5].

The DEL, DHL, and THL incidences vary. Huang and colleagues described an incidence of 7.7% for DHL/THL [3] while Ma and colleagues found 14% of DHL and 34% of DEL in their DLBCL series [6]. It is generally estimated that around 20 to 30% of patients diagnosed with DLBCL have an overexpression of *MYC* and *BCL2* or *BCL6* [2,7].

Standard treatment for DLBCL is R-CHOP (rituximab, cyclophosphamide, doxorubicin, vincristine, and prednisone) and, while current guidelines suggest intensified chemotherapy protocols for DHL and THL (mostly R-DA-EPOCH—dose-adjusted rituximab, etoposide, cyclophosphamide, doxorubicin, vincristine, and prednisone) [8], the evidence to intensively treat DEL is weak, based on a small number of patients enrolled and the retrospective design of the studies [9,10]. DEL has worse progression-free survival (PFS) and OS independent of the international prognostic index (IPI) and cell-of-origin (COO) [11] and is considered an adverse prognostic indicator in DLBCL [1]. Yet, some retrospective series report a favorable outcome for limited-stage patients treated with R-CHOP [12]. A German high-grade lymphoma study group performed a post hoc analysis of DLBCL patients, treated in prospective clinical trials, regarding their COO and expression of *MYC*, *BCL2*, and *BCL6*. Their analysis indicated that, in patients treated with R-CHOP, the double-expressor (DE) status was associated with significantly inferior survival compared to patients without DE status; however, this is only in the germinal center B-cell (GCB) COO subgroup [13]. As the IPI score has been confirmed as a useful prognostic tool for patients with DLBCL [14], it has been more recently established, also, as an independent prognostic factor for DHL and DEL [6].

The aim of this study was to evaluate the incidence of DHL, THL, and DEL in our real-world consecutive series of DLBCL patients and evaluate their clinical characteristics as well as their first-line treatment regimens. Moreover, we wanted to compare survival among patients treated with standard R-CHOP and those treated with more intensive chemotherapy protocols.

## 2. Materials and Methods

### 2.1. Patients

A retrospective analysis of all diffuse large B-cell lymphomas and high-grade B-cell lymphomas with *MYC* and *BCL2* and/or *BCL6* rearrangements, diagnosed between 2017 and 2021 at the Institute of Oncology Ljubljana (IOL), Slovenia, has been performed. Only patients with DEL, DHL, or THL were included in the present study. Patients treated with at least one cycle of systemic treatment were included in the analyses while those fit only for palliative radiotherapy or supportive treatment were excluded. Patients whose histological specimens were referred to our institute for consultation purposes only but have been treated elsewhere were also excluded. Data regarding the patients’ age, clinical stage, laboratory values, IPI score, treatment protocols, and outcomes were obtained from the hospital’s information system. Only treatment regimens applied for DEL/DHL/THL were noted and analyzed for the purpose of this article. In case DEL/DHL/THL was diagnosed at the relapse of a previously non-DEL/DHL/THL condition, the treatment protocol used in this relapse setting was noted and acknowledged as a first-line treatment of DEL/DHL/THL.

Survival data were retrieved from the Cancer Registry of the Republic of Slovenia and survival status was censored for all patients on 9 May 2022.

This study was approved by the Institutional Review Board (approval number KSOPKR-0088/2021) and the Institutional Medical Ethics Committee (approval number EK-0100/2021), which waived the informed consent for each individual patient as this was a retrospective database analysis and the institutional informed consent form for treatment included consent to use the patient’s data, materials, and/or test results for research purposes. This study was conducted according to the Declaration of Helsinki.

### 2.2. Pathohistological Examination

All lymph node biopsies that were, at the time of the diagnosis, routinely fixed in 10% buffered formalin for 24 h and stained by H&E; evaluated immunohistochemically (on Benchmark XT Ventana Medical Systems, Inc., Tucson, AZ, USA) for CD20 (1:50, clone, L26; Dako, Agilent Technologies, Inc., Glostrup, Danmark), CD3 (1:50, Clone F7.2.38, Dako, Agilent Technologies, Inc., Glostrup, Danmark), CD5 (1:400, clone, 4C7; Novocastra, Leica Microsystems, Inc., Wetzlar, Germany), CD10 (1:20, clone, 56C6; Novocastra, Leica Microsystems, Inc., Wetzlar, Germany), BCL-6 (clone, GI 191E/A8; Cell Marque, Sigma-Aldrich, Merck KGaA, Darmstadt, Germany), BCL-2 (1:40,clone 124; Dako, Agilent Technologies, Inc., Glostrup, Denmark), CD23 (1:100, Clone DAK-CD23, Dako, Agilent Technologies, Inc., Glostrup, Denmark), CD21 (1:200, Clone 1F8, Dako, Agilent Technologies, Inc., Glostrup, Denmark), c-MYC (1:200, Clone 9E10, Dako, Agilent Technologies, Inc., Glostrup, Denmark), MUM-1 (1:100, clone, MUM1p; Dako, Agilent Technologies, Inc., Glostrup, Denmark), cycline D1 (1:50, clone DCS-6, Sigma Aldrich, Burlington, Massachusetts, USA), and MIB-1 (1:200, Ki67; clone, MIB1; Dako, Agilent Technologies, Inc., Glostrup, Denmark) and evaluated by FISH (DNA break apart probes Dako MYC (8q24), Dako BCL2 (18q21) and Dako BCL6 (3q27); Agilent Technologies, Inc., Glostrup, Denmark) when needed were retrieved from the archive of the Department of the Pathology of the Institute of Oncology Ljubljana and reviewed independently by two skilled hematopathologists who were blinded for any clinical information. After the revision, the results were compared and all discrepant cases were discussed further under the multi-head microscope. The DLBCLs were subdivided into GCB and non-GCB (ABC) subtypes according to the Hans algorithm [15]. Cases with MYC and BCL2 overexpression in ≥40% and >50% of lymphoma cells, respectively, were categorized as DEL. Cases with proven *MYC* and *BCL2* or *BCL6* translocation by FISH were categorized as DHL and those with rearrangements of all mentioned genes as THL.

### 2.3. Statistical Analyses

Descriptive statistics were made for demographic data and the Mann–Whitney test was applied for numeric variables. Survival was estimated using the Kaplan–Meier survival method and differences were compared using the log-rank tests. Overall survival was defined as the time interval from diagnosis until death from any cause. Progression-free survival was defined as the time interval from the end of the first-line treatment until progression or death from any cause for those achieving partial or complete remission. The Cox proportional hazards regression model was used for multivariate analysis and a two-sided *p* value less than 0.05 was considered statistically significant. Statistical analyses were performed using IBM SPSS Statistics, version 26.

## 3. Results

### 3.1. Demographic Data

Altogether, 309 cases of DLBCL and 22 cases of HGBL (either NOS or with *MYC* and *BCL2* and/or *BCL6* rearrangements) were diagnosed in the period from 2017 to 2021 at the IOL. Additionally, 161 out of a total of 309 DLBCL cases (161/309; 52,1%) were classified as DEL. Sixteen patients had DHL (16/331 whole cohort—4.8%), *MYC/BCL2* rearrangement was observed in eleven patients, and *MYC/BCL6* rearrangement in five patients. Five patients were diagnosed with THL (5/331 whole cohort—1.5%). After the recognition of the above-mentioned inclusion and exclusion criteria (patients fit only for palliative radiotherapy or supportive treatment and those treated elsewhere were excluded), 154 cases underwent further analyses, 79 males (51.3%) and 75 females (48.7%).

Out of one-hundred and fifty-four patients included, one-hundred and thirty-five patients had DEL, sixteen patients had DHL, and three patients had THL. Out of 135 DELs, 80 patients were classified as having the ABC subtype and 55 patients as having the GCB subtype according to the Hans algorithm [15]. Sixteen patients with the GCB subtype in whom CD10 was positive also displayed a positive expression of MUM1 in >50% of lymphoma cells and were classified as the so-called “double positive” type (MUM1 and CD10).

Demographic and descriptive data of the included patients are presented in Table 1.

### 3.2. Treatment

Patients were divided into four therapeutic groups for the purpose of the analyses—Group A, which received R-CHOP (or R-mini-CHOP in older patients); Group B, which received R-CHOP with intermediate-dose methotrexate (0.5 g/m^2^); Group C, which received R-DA-EPOCH; and Group D, which received a therapeutic regimen without anthracyclines (the reasons being pre-existing cardiac comorbidity, previous anthracycline exposure and exceeded cumulative dose, or relapsed disease where DEL/DHL was diagnosed at relapse and not as the first lymphoma diagnosis). In the case of switching the therapy in a first-line setting, patients were appointed to the treatment group based on preponderance of the applied regimen. Therapeutic groups according to the number of patients are given in Table 2 and treatment parameters in Table 3.

According to ESMO Guidelines [16], central nervous system (CNS) prophylaxis was administered to 78 patients (50.7%), either by intrathecal chemotherapy with methotrexate and cytarabine (75/78) or intravenously with high dose methotrexate (3/78). Eight patients (8/154—5.2%) experienced CNS relapse; three of them received no CNS prophylaxis.

### 3.3. Survival

At the time of data acquisition, 105 patients were still alive (68.2%). Out of 49 deceased patients, 37 died due to lymphoma or lymphoma-related causes (75.5%) and 12 due to other causes (24.5%), such as advanced age, suicide, or car accident. The median follow-up time was 22 months.

Altogether, 110 patients had no relapse in the observation period (71.4%), 34 progressed during or after first-line treatment for DEL/DHL/THL (21.1%), and 10 patients could not be evaluated. Out of thirty-four relapsed patients, twenty-six patients (76.5%) had a systemic relapse and eight had a CNS relapse (23.5%). Fourteen patients relapsed after achieving a CR.

#### 3.3.1. Double-Expressor Group

Descriptive parameters of the double-expressor group are given in Table 4. Thirty-seven patients died during follow-up, the majority (68%) due to lymphoma progression.

**Figure 1:**
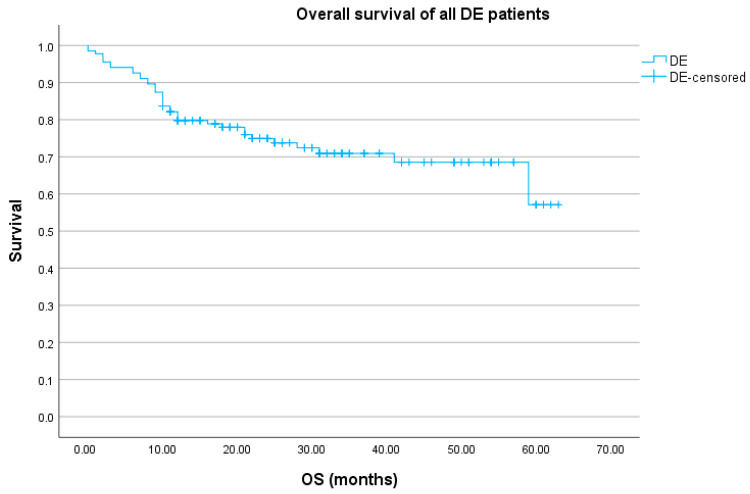
OS of all DE DLBCL patients (N = 122).

**Figure 2:**
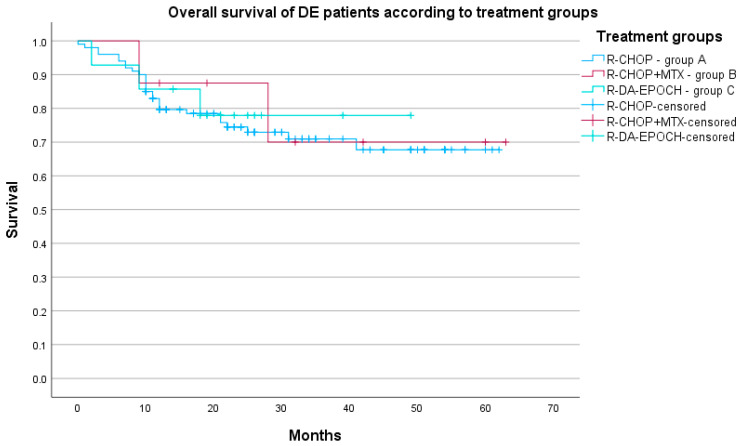
OS of DE DLBCL patients (N = 122) according to three treatment groups (*p* = 0.939).

The five-year OS for the whole DEL group was 57.1% (95% CI 45.9%–68.3%) and the 5-year PFS was 76.5% (95% CI 72.6%–80.4%). Survival analyses were made according to four treatment groups; however, due to the fact that the 5-year OS for Group D was 0%, we display results at a 3-year point: the 3-year OS for Group A was 71.0% (95% CI 66.0–76.0%), 70.0% for Group B (95% CI 51.8–88.2%), 77.9% for Group C (95% CI 66.6–89.2%), and 69.2% for Group D (95% CI 56.4–82.0%). The log-rank test displayed no differences in survival between groups (*p* = 0.712). The overall survival of the whole group is given in Figure 1 and OS according to treatment groups is given in Figure 2.

**Figure 3:**
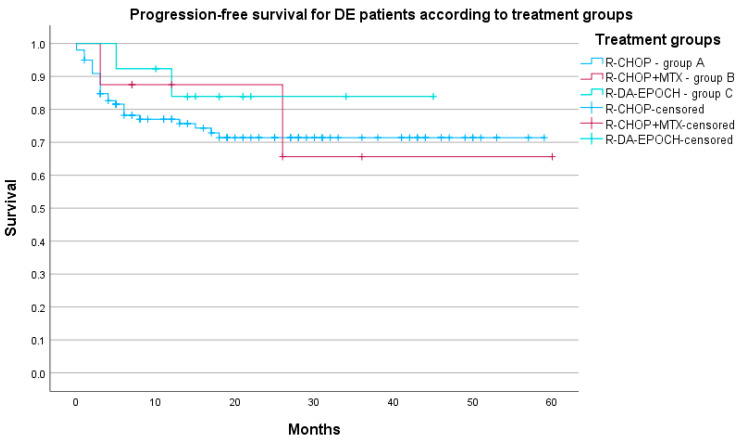
PFS of DE DLBCL patients (N = 122) according to three treatment groups (*p* = 0.674).

Three-year PFS was 71.4% for Group A (95% CI 66.6–76.2%), 65.6% for Group B (95% CI 44.7–86.5%), 83.9% for Group C (95% CI 73.5–94.3%), and 80.8% for Group D (95% CI 68.6–93.0%). The log-rank test detected no differences in PFS between groups (*p* = 0.843). Progression-free survival according to treatment groups is presented in Figure 3.

Regarding the COO, as determined by the Hans algorithm, there was no difference in OS or PFS between the ABC (80 patients) and GCB (55 patients) subtypes, *p* = 0.681 and *p* = 0.297, respectively. The 3-year OS for the ABC subgroup was 66.8% (95% CI 60.8–72.8%) and for the GCB subgroup was 77.1% (95% CI 71.2–83.0%). The 3-year PFS for the ABC subgroup was 68.3% (95% CI 62.4–74.2%) and for the GCB subgroup was 79.4% (95% CI 73.5–85.3%).

**Figure 4:**
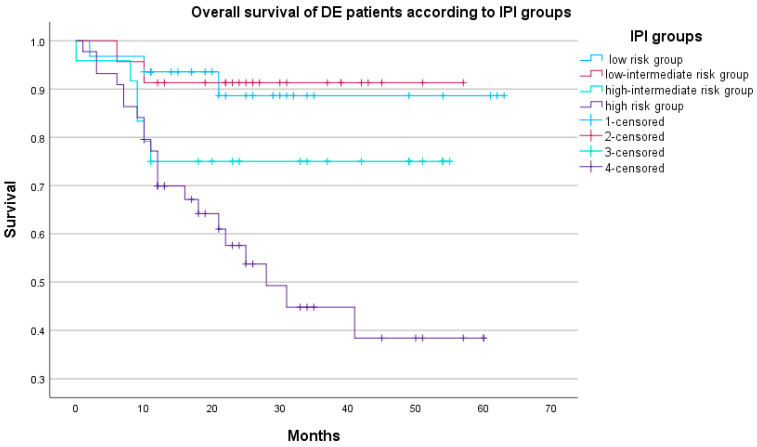
OS according to IPI groups (N = 122), *p* = 0.003.

**Figure 5:**
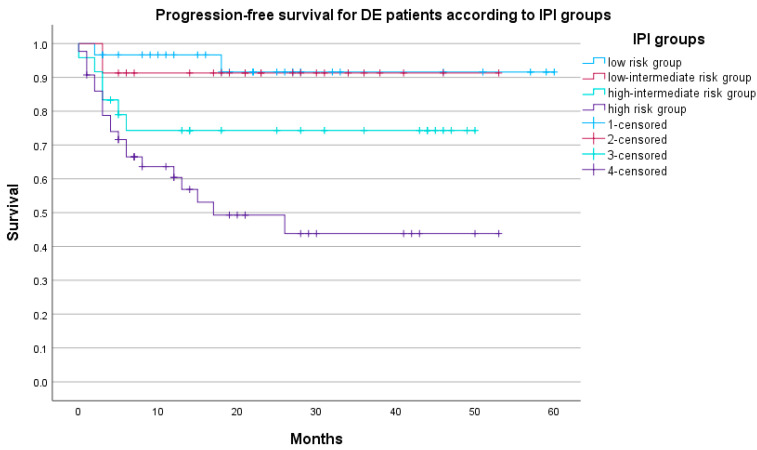
PFS according to IPI groups (N = 122), *p* = 0.003.

When comparing the survival of DEL patients among different IPI risk groups, there was a significantly higher risk of death in the high-risk IPI group (HR 7.68, 95% CI 2.32–25.49, *p* = 0.001) while the risk of death for other IPI risk groups was not statistically significantly different (low vs. high-intermediate group *p* = 0.993, low-intermediate vs. high-intermediate group *p* = 0.268). The OS according to the IPI risk groups is given in Figure 4. As for the PFS, a significant difference was found for the high-intermediate IPI risk group (HR 5.48, 95% CI 1.13–26.64, *p* = 0.035) and for the high-risk IPI group (HR 13.26, 95% CI 2.97–59.25, *p* = 0.001) while the risk of progression or death for the other two IPI risk groups was not statistically significantly different (low vs. high-intermediate group *p* = 0.604, low-intermediate vs. high-intermediate group *p* = 0.305). The PFS according to the IPI groups is presented in Figure 5.

A comparison of cases according to MUM1 expression has also been performed with the cut-off for a higher expression of >50% in the GCB group. The three-year OS for the high-expression group was 73.3% (61.9%–84.7%) and it was 78.4% (71.5–85.3%) for the low-expression group, respectively, and the difference was insignificant, *p* = 0.688. There was also no difference in 3-year PFS between groups; the values were 91.7% (83.7–99.7%) and 81.1% (74.0–88.2%) for the high- and low-expression groups, respectively (*p* = 0.432).

#### 3.3.2. Double- and Triple-Hit Group

This group consisted of nineteen patients only, sixteen with DHL and three of them having a THL. The median age of the DHL patients was 69 years (range 23–87 years) and it was 66 years for THL (range 58–79 years). Eleven DHL and all three THL patients were male. Eight DHL patients (50.0%) remained in remission after first-line treatment for DHL during the follow-up period and five patients relapsed while three could not be evaluated.

The five-year OS for DHL patients was 32.4% (95% CI 16.6–48.2%); all three TH patients were deceased or lost to follow-up. The five-year PFS for DHL patients was 34.9% (95% CI 18.1–53.0%). Analyses according to treatment groups and IPI risk groups were not conducted due to a small sample size and, therefore, a weak statistical power.

## 4. Discussion

This is a retrospective analysis of real-world patients with the DEL subtype of DLBCL and DHL/THL, treated with different therapeutic protocols.

Regarding DHL and THL proportions and median age, our group did not differ much from the published literature. Landsburg and colleagues reported approximately 65% of patients categorized as DHL to have translocations of *MYC* and *BCL2*, 14% to have translocations of *MYC* and *BCL6*, and the remaining 21% to have all three translocations [17]. Our study revealed a roughly similar distribution—57.9% of DHL patients had *MYC*/*BCL2*, 26.3% had *MYC*/*BCL6* rearrangements, and the remaining 15.8% had all three translocations. As well, the median age of 70 years is in line with the study of Green and colleagues [11]. However, regarding other baseline characteristics, our group had lower IPI scores, lower LDH concentrations, and a lesser percentage of patients with extranodal involvement compared to previously published studies [2], which are all known factors that impact patients’ prognosis. This distribution of prognostic features in our study group could have partially contributed to the better survival rate among our DEL patients compared to the literature, even though it probably cannot be fully ascribed to it since this is a consecutive group of all patients admitted to our institute. The lower IPI scores in our study group could as well potentially offer an explanation for a relatively high percentage of patients achieving a CR after the first-line treatment—a percentage that was higher than in the study by Green and colleagues (82.2% in our study and 73% in their study) [11].

According to the literature, there is some evidence that patients in whom DLBCL is CD10 positive (GCB type) and who have a high MUM-1 expression in >50% of lymphoma cells have a worse prognosis, similar to the prognosis of the ABC subtype [18]. This was, however, not confirmed in our series of patients since we observed no difference in the survival of the GCB subgroup regarding high MUM1 expression.

We determined a rather high proportion of DEL among DLBCL (52.1%), which can be partially attributed to the fact that our pathologists adhere to the WHO recommendation of a 50% cut-off for BCL2 positivity. A recently published large meta-analysis by Hwang and colleagues [7] gathered a number of studies regarding DLBCL and DEL/DHL and demonstrated that only 51% of involved studies applied the cut-off of 50% for BCL2 positivity; meanwhile, in other studies, the cut-off values were usually higher (70% being the most prevalent cut-off for the BCL2 staining). And, including all those studies and adjusting for the publication bias and heterogeneity, their pooled proportion of DEL among DLBCL was 31% (95% CI 27–36%).

Nevertheless, the survival of DHL patients was unsurprisingly low and comparable to previous reports from the literature [2,19] while the survival of our DEL group was unexpectedly high. The PFS and OS rates of our DEL patients were, namely, in line with rates previously published from our center for a series of standard DLBCL patients treated with R-CHOP [20]. The PFS rate of 77% and OS rate of 57% do not differ importantly from the rates published in 2018, being 80% and 63% for PFS and OS, respectively [20], leading to the conclusion that the survival of our DEL patients is quite similar to the survival of the whole (standard) DLBCL group of patients. Still, a direct head-to-head comparison cannot be made since the DEL patients were not evaluated separately in the previous study. Furthermore, survival rates in the present study were rather high in all treatment subgroups and we found no differences between the treatment groups. Since these groups consisted of real-world patients and not balanced randomized trial groups, the distribution of patients was definitely uneven. Nevertheless, we do believe that these data raise a justifiable question of the necessity of the treatment of DEL with more intensive treatment protocols compared to R-CHOP. While treatment recommendations for DHL and THL incline towards intensified treatment protocols (e.g., R-DA-EPOCH) versus R-CHOP, which also yields better survival with R-DA-EPOCH [21], the literature also recommends intensified regimens to overcome the dire prognostic impact of the DE phenotype. Still, these data are based on younger and smaller series of patients [9,10]. Accordingly, limited reports have already been published comparing the R-CHOP and R-DA-EPOCH in the DEL patient population, among which is a study by Devi and colleagues [22] that included 113 patients and a multicentre study of 90 patients in the USA [23]. Both studies actually disclosed no differences in survival among the two protocols applied. Finally, in the study by Zhang and colleagues, the sub-analysis of 53 DEL patients determined that the R-DA-EPOCH is most probably incapable of overcoming the poor prognosis of the DE phenotype compared to the R-CHOP protocol [24]. To the best of our knowledge, our study represents the one with the largest number of DEL patients being treated at the same center; therefore, all patients received equal or at least equivalent medical management. It is, therefore, quite interesting that we found no difference in the OS or PFS in regard to the COO in the DEL patients as the ABC subtype is known for its worse prognosis. However, a possible explanation for this observation can be the fact that the current ABC and GCB subgroups provide too little clinical utility because they do not take into account the numerous changes that can occur within the genome that are not observable with immunohistochemical staining techniques but are impactful to the overall treatment outcome. It has been also shown that certain genetic subtypes observed predominately among the GCB subgroup (i.e., EZB subtype) do not share the good prognosis of the entire GCB subgroup and that certain genetic subtypes observed predominately among the ABC subgroup (i.e., BN2 subtype) do not share the poor prognosis of the entire ABC subgroup [25]. On the other hand, we shall also not ignore that some recent studies reported no differences in survival regarding the COO for patients without DEL as well [12,13]. Nevertheless, we managed to confirm, once again, that high-risk IPI features in the DEL group convey the worse prognosis and that those patients experience more relapses than patients in more favorable IPI groups.

The advantage of this study is that these are real-world patients who are not biased by a selection for the clinical trial and who were treated in a real-world setting with their comorbidities and complications and given performance statuses as chosen by the treating physician. All histopathological specimens were also centrally reviewed by two skilled hematopathologists. The disadvantages, conversely, are the unequal numerical distribution across the treatment groups, with the most widely represented R-CHOP treatment, and, unquestionably, the retrospective methodology of this study. Still, this comparison without significant differences in the survivals of the DEL patients is encouraging, bringing the perspective that not all patients with DEL require intensive treatment protocols and can be safely treated with standard R-CHOP.

## 5. Conclusions

The significance of our study is that the R-CHOP protocol with CNS prophylaxis (when indicated) has been proven a successful and curative treatment in a substantial proportion of real-world DEL patients who were not biased by a selection for the clinical trial and who were treated in a real-world setting as chosen by the treating physician. In these patients, excessively toxic intensive protocols, such as R-DA-EPOCH, could most probably be safely replaced with standard R-CHOP. Considering that this is not a randomized study, our results should be confirmed in a larger prospective randomized trial aiming also to identify those DEL patients who should (due to a higher risk, potentially attributable to high-risk IPI) receive a more intensive treatment.

At present, with a lack of a more profound routine molecular testing of the DLBCL, and considering the above-stated results, there is no justified recommendation to treat the DEL with a more intensive treatment than R-CHOP.

## Figures and Tables

**Table 1:** Demographic and descriptive data for all included patients, the DE subgroup, and the DHL/THL subgroup. LDH—lactate dehydrogenase, IPI—international prognostic index.

Number of Patients	The Whole Cohort (N = 154)	DE DLBCL Patients (N = 122)	DHL/THL Patients (N = 16)
Male/Female	79 (51.3%)/75 (48.7%)	60 (49.2%)/62 (50.8%)	11 (68.8%)/5 (31.2%)
Median age at diagnosis	70 years (range 21–91 years)	70 years (range 21–89 years)	67 years (range 23–87 years)
Median stage at diagnosis	4 (range 1–4)	4 (range 1–4)	4 (range 1–4)
Constitutional symptoms	66 patients (42.9%)	54 patients (44.3%)	9 patients (56.2%)
Bulky disease	28 patients (18.2%)	20 patients (16.4%)	5 patients (31.2%)
Extranodal involvement	74 patients (48.0%)	56 patients (45.9%)	9 patients (56.2%)
Elevated LDH level	86 patients (55.8%)	68 patients (55.7%)	12 patients (75.0%)
Low-risk IPI score (0, 1 points)	39 patients (25.4%)	31 patients (25.4%)	4 patients (25.0%)
Low-intermediate-risk IPI score (2 points)	27 patients (17.5%)	23 patients (18.8%)	1 patient (6.2%)
High-intermediate-risk IPI score (3 points)	31 patients (20.1%)	24 patients (19.7%)	4 patients (25.0%)
High-risk IPI score (4, 5 points)	57 patients (37.0%)	44 patients (36.1%)	7 patients (43.8%)

**Table 2:** Therapeutic groups of patients. R-CHOP—rituximab, cyclophosphamide, doxorubicin, vincristine, and prednisone; MTX—methotrexate; R-DA-EPOCH—dose-adjusted rituximab, etoposide, cyclophosphamide, doxorubicin, vincristine, and prednisone; R-COEP—rituximab, cyclophosphamide, etoposide, vincristine, and prednisone; GemOx—gemcitabine and oxaliplatin; R-CBVPP—rituximab, cyclophosphamide, carmustine, procarbazine, and prednisone.

Therapeutic Group	Therapeutic Regimen Used	Number of Patients (N = 154)	DE DLBCL Patients (N = 122)	DHL/THL Patients (N = 16)
Group A	R-CHOP	109 (70.8%)	100 (81.9%)	9 (58.8%)
Group B	R-CHOP + intermediate-dose MTX	10 (6.5%)	8 (6.6%)	2 (11.8%)
Group C	R-DA-EPOCH	19 (12.3%)	14 (11.5%)	5 (29.4%)
Group D	R-COEP, GemOx, R-CBVPP	16 (10.4%)	13 (excluded from the 135-patient primary group)	3 (excluded from the 19-patient primary group)

**Table 3:** Treatment parameters of the included patients, DE DLBCL patients, and DHL/THL patients. In the DE DLBCL and DHL/THL subgroups, only patients from Treatment Groups A, B, and C are included. †—the treatment outcome was not evaluated due to complications of treatment, loss of follow-up, or the patient’s refusal to finish the treatment or perform objective treatment evaluation.

	All Patients (N = 154)	DE DLBCL Patients (N = 122)	DHL/THL Patients (N = 16)
Median number of cycles	6 (range 1–8)	6 (range 1–8)	6 (range 1–8)
Radiotherapy consolidation	59 (59/154 = 38.3%)	46 (46/122 = 37.7%)	7 (7/16 = 43.8%)
Outcome: complete remission (CR)	118 (118/154 = 76.6%)	101 (101/122 = 82.8%)	9 (9/16 = 56.4%)
Outcome: partial remission (PR)	6 (6/154 = 3.9%)	4 (4/122 = 3.3%)	1 (1/16 = 6.2%)
Outcome: stable disease (SD)	1 (1/154 = 0.7%)	0 (0%)	0 (0%)
Outcome: progressive disease (PD)	19 (19/154 = 12.3%)	13 (13/122 = 10.6%)	3 (3/16 = 18.7%)
Treatment outcome was not evaluated †	10 (10/154 = 6.5%)	4 (4/122 = 3.3%)	3 (3/16 = 18.7%)

**Table 4:** Therapeutic and other parameters of the DE DLBCL group of patients. R-CHOP—rituximab, cyclophosphamide, doxorubicin, vincristine, and prednisone; MTX—methotrexate; R-DA-EPOCH—dose-adjusted rituximab, etoposide, cyclophosphamide, doxorubicin, vincristine, and prednisone; CNS—central nervous system.

Therapeutic Group	Therapeutic Regimen	Number of DE DLBCL Patients (N = 122)	Median IPI Value	Median Age at Diagnosis	Received CNS Prophylaxis	Progression of Lymphoma	Progression into CNS	Died/Due to Lymphoma
Group A	R-CHOP	100	3	70 (range 21–89)	44 (44/100 = 44.0%)	20 (20/100 = 20.0%)	6 (6/20 = 30.0%)	27 (27/100 = 27.0%)/19 (19/27 = 70.4%)
Group B	R-CHOP + intermediate-dose MTX	8	3.5	64 (range 38–71)	8 (8/8 = 100%)	2 (2/8 = 25.0%)	1 (1/2 = 50.0%)	2 (2/8 = 25.0%)/1 (1/2 = 50.0%)
Group C	R-DA-EPOCH	14	3	57 (range 48–75)	9 (9/14 = 64.3%)	2 (2/14 = 14.3%)	0	3 (3/14 = 21.4%)/3 (3/3 = 100%)

## Data Availability

Data are stored and can be obtained upon request by the corresponding author.

## References

[1] Swerdlow S.H., Campo E., Pileri S.A., Harris N.L., Stein H., Siebert R., Advani R., Ghielmini M., Salles G.A., Zelenetz A.D. (2016). The 2016 revision of the World Health Organization classification of lymphoid neoplasms. Blood.

[2] Riedell P.A., Smith S.M. (2018). Double hit and double expressors in lymphoma: Definition and treatment. Cancer.

[3] Huang S., Nong L., Wang W., Liang L., Zheng Y., Liu J., Li D., Li X., Zhang B., Li T. (2019). Prognostic impact of diffuse large B-cell lymphoma with extra copies of MYC, BCL2 and/or BCL6: Comparison with double/triple hit lymphoma and double expressor lymphoma. Diagn. Pathol..

[4] Roh J., Cho H., Pak H.-K., Lee Y.S., Lee S.-W., Ryu J.-S., Chae E.J., Kim K.W., Huh J., Choi Y.S. (2022). BCL2 super-expressor diffuse large B-cell lymphoma: A distinct subgroup associated with poor prognosis. Mod. Pathol..

[5] Alaggio R., Amador C., Anagnostopoulos I., Attygalle A.D., Araujo I.B.d.O., Berti E., Bhagat G., Borges A.M., Boyer D., Calaminici M. (2022). The 5th edition of the World Health Organization Classification of Haematolymphoid Tumours: Lymphoid Neoplasms. Leukemia.

[6] Ma Z., Niu J., Cao Y., Pang X., Cui W., Zhang W., Li X. (2020). Clinical significance of ‘double-hit’ and ‘double-expression’ lymphomas. J. Clin. Pathol..

[7] Hwang J., Suh C.H., Kim K.W., Kim H.S., Kim A.I., Craig J.W., Chen K.X., Roberson J., Guenette J.P., Huang R.Y. (2021). The Incidence and Treatment Response of Double Expression of MYC and BCL2 in Patients with Diffuse Large B-Cell Lymphoma: A Systematic Review and Meta-Analysis. Cancers.

[8] Zelenetz A.D., Gordon L.I., Abramson J.S., Advani R.H., Andreadis B., Bartlett N.L., Budde L.E., Caimi P.F., Chang J.E., Christian B. (2022). NCCN Guidelines: B-cell Lymphomas, Version 5. www.nccn.org.

[9] Pedersen M.Ø., Gang A.O., Brown P., Pedersen M., Knudsen H., Nielsen S.L., Poulsen T., Klausen T.W., Høgdall E., Nørgaard P. (2017). Real world data on young patients with high-risk diffuse large B-cell lymphoma treated with R-CHOP or R-CHOEP—MYC, BCL2 and BCL6 as prognostic bio-markers. PLoS ONE.

[10] Dodero A., Guidetti A., Tucci A., Barretta F., Novo M., Devizzi L., Re A., Passi A., Pellegrinelli A., Pruneri G. (2019). Dose-adjusted EPOCH plus rituximab improves the clinical outcome of young patients affected by double expressor diffuse large B-cell lymphoma. Leukemia.

[11] Green T.M., Young K.H., Visco C., Xu-Monette Z.Y., Orazi A., Go R.S., Nielsen O., Gadeberg O.V., Mourits-Andersen T., Frederiksen M. (2012). Immunohistochemical Double-Hit Score Is a Strong Predictor of Outcome in Patients with Diffuse Large B-Cell Lymphoma Treated with Rituximab Plus Cyclophosphamide, Doxorubicin, Vincristine, and Prednisone. J. Clin. Oncol..

[12] Barraclough A., Alzahrani M., Ettrup M.S., Bishton M., van Vliet C., Farinha P., Gould C., Birch S., Sehn L.H., Sovani V. (2019). COO and MYC/BCL2 status do not predict outcome among patients with stage I/II DLBCL: A retrospective multicenter study. Blood Adv..

[13] Staiger A.M., Ziepert M., Horn H., Scott D.W., Barth T.F., Bernd H.-W., Feller A.C., Klapper W., Szczepanowski M., Hummel M. (2017). Clinical Impact of the Cell-of-Origin Classification and the *MYC*/*BCL2* Dual Expresser Status in Diffuse Large B-Cell Lymphoma Treated Within Prospective Clinical Trials of the German High-Grade Non-Hodgkin’s Lymphoma Study Group. J. Clin. Oncol..

[14] Shipp M.A. (1993). International Non-Hodgkin’s Lymphoma Prognostic Factors Project. A predictive model for aggressive non-Hodgkin’s lymphoma. N. Engl. J. Med..

[15] Hans C.P., Weisenburger D.D., Greiner T.C., Gascoyne R.D., Delabie J., Ott G., Müller-Hermelink H.K., Campo E., Braziel R.M., Jaffe E.S. (2004). Confirmation of the molecular classification of diffuse large B-cell lymphoma by immunohistochemistry using a tissue microarray. Blood.

[16] Hutchings M., Ladetto M., Buske C., de Nully Brown P., Ferreri A.J.M., Pfreundschuh M., Schmitz N., Sureda Balari A., van Imhoff G., Walewski J. (2018). ESMO Consensus Conference on Malignant Lymphoma: Management of ‘ultra-high-risk’ Patients. Ann. Oncol..

[17] Landsburg D.J., Petrich A.M., Abramson J.S., Sohani A.R., Press O., Cassaday R., Chavez J.C., Song K., Zelenetz A.D., Gandhi M. (2016). Impact of oncogene rearrangement patterns on outcomes in patients with double-hit non-Hodgkin lymphoma. Cancer.

[18] Irawan C., Iskandar M., Harahap A.S., Rumende C.M., Ham M.F. (2022). MUM1 Expression versus Hans Algorithm to Predict Prognosis in Indonesian Diffuse Large B-Cell Lymphoma Patients Receiving R-CHOP. Cancer Manag. Res..

[19] Cortese M.J., Wei W., Cerdeña S., Watkins M.P., Olson M., Jodon G., Kaiser J., Haverkos B., Hughes M.E., Namoglu E. (2023). A multi-center analysis of the impact of DA-EPOCH-R dose-adjustment on clinical outcomes of patients with double/triple-hit lymphoma. Leuk. Lymphoma.

[20] Horvat M., Zadnik V., Šetina T.J., Boltežar L., Goličnik J.P., Novaković S., Novaković B.J. (2018). Diffuse large B-cell lymphoma: 10 years’ real-world clinical experience with rituximab plus cyclophosphamide, doxorubicin, vincristine and prednisolone. Oncol. Lett..

[21] Goyal G., Magnusson T., Wang X., Roose J., Narkhede M., Seymour E. (2023). Modern, real-world patterns of care and clinical outcomes among patients with newly diagnosed diffuse large B-cell lymphoma with or without double/triple-hit status in the United States. Haematologica.

[22] Devi K., Shaikh M.U., Ali N.B., Adil S.N., Khan M., Soomar S.M. (2021). Outcomes of patients with double/triple expressor diffuse large B-cell lymphoma (DLBCL) treated with R-DA-EPOCH/R-CHOP: A single-center experience. Leuk. Res. Rep..

[23] D’Angelo C.R., Hanel W., Chen Y., Yu M., Yang D., Guo L., Karmali R., Burkart M., Ciccosanti C., David K. (2021). Impact of initial chemotherapy regimen on outcomes for patients with double-expressor lymphoma: A multi-center analysis. Hematol. Oncol..

[24] Zhang X.-Y., Liang J.-H., Wang L., Zhu H.-Y., Wu W., Cao L., Fan L., Li J.-Y., Xu W. (2019). DA-EPOCH-R improves the outcome over that of R-CHOP regimen for DLBCL patients below 60 years, GCB phenotype, and those with high-risk IPI, but not for double expressor lymphoma. J. Cancer Res. Clin. Oncol..

[25] Schmitz R., Wright G.W., Huang D.W., Johnson C.A., Phelan J.D., Wang J.Q., Roulland S., Kasbekar M., Young R.M., Shaffer A.L. (2018). Genetics and Pathogenesis of Diffuse Large B-Cell Lymphoma. N. Engl. J. Med..

